# Prognostic value of preoperative CCL26 serum levels in the surgical treatment of chronic rhinosinusitis

**DOI:** 10.1590/1414-431X2026e14736

**Published:** 2026-08-07

**Authors:** Xudong Wang, Shunan Liu, Jichuan Chen, Wanting Zeng, Jianguo Jiang

**Affiliations:** 1 Otorhinolaryngology Department, Daping Hospital of Army Medical University, PLA, Chongqing, China

**Keywords:** Chronic rhinosinusitis, Eotaxin, CCL26, Bioinformatics, SNOT22

## Abstract

Eosinophilic chronic rhinosinusitis (CRS), characterized by prominent eosinophil infiltration, has the highest postoperative recurrence rate among CRS subtypes. Given the critical role of eotaxins in CRS pathogenesis, this study aimed to identify eotaxins associated with eosinophilic CRS and evaluate their correlation with surgical outcomes. Transcriptomic data from GSE36830 revealed 125 differentially expressed genes (49 up-regulated, 76 down-regulated) in CRSwNP compared to CRSsNP, with significant enrichment of eosinophil and dendritic cell infiltration pathways. Functional analysis identified chemotaxis and cell migration as key dysregulated processes in CRSwNP. Among eotaxins, CCL26 and CCL13 emerged as hub molecules from the intersection of these dysregulated pathways. In a clinical cohort of 115 CRS patients, preoperative serum CCL26 and CCL13 levels were significantly elevated in CRSwNP compared to CRSsNP (P<0.001). ROC analysis demonstrated strong diagnostic power for nasal polyps (CCL26: AUC=0.895; CCL13: AUC=0.830). Higher preoperative CCL26 levels predicted poorer symptom improvement post-surgery (standardized β=−0.564, P<0.001). In conclusion, preoperative serum CCL26 serves as both a diagnostic biomarker for CRSwNP and a prognostic indicator for surgical outcomes, with elevated levels associated with reduced symptom improvement.

## Introduction

Chronic rhinosinusitis (CRS) is a chronic inflammatory disease that occurs in the mucous membrane of the nasal cavity and sinuses with a duration of more than 12 weeks. Epidemiologic surveys have shown that the overall prevalence of CRS in Chinese people is 8% (1), which is higher than that in Brazil, Korea, and Canada (2). Its clinical symptoms are mainly manifested as nasal congestion, mucopurulent mucus, dizziness, headache, olfactory dysfunction, and memory loss. The symptoms of the disease directly affect the patient's daily life, resulting in reduced work ability, social activity, and quality of life and increased economic burden. According to a survey in the United States, CRS affects 14-16% of the population, costing about 5.7 billion dollars a year (3). In addition, insufficient pulmonary ventilation related to chronic nasal congestion can lead to chronic hypoxia and obstructive sleep apnea syndrome, which may gradually lead to hypertension, diabetes mellitus, and other systemic diseases (4).

According to the European clinical guidelines (EPOS-2012) and the Chinese clinical guidelines (CPOS-2018), CRS can be classified into chronic rhinosinusitis without nasal polyps (CRSsNP) and chronic rhinosinusitis with nasal polyps (CRSwNP). Patients with CRS are usually first treated with pharmacologic therapy based on nasal glucocorticoids. Those who do not respond to the treatment may undergo functional endoscopic sinus surgery (FESS). However, even after receiving the standardized treatment recommended by the guidelines, there are still a considerable number of patients that do not achieve satisfactory outcomes. International studies have shown that the recurrence rate of CRS patients 12 years after surgery is 20-80% (5). Approximately 29% of Chinese CRS patients have difficult-to-treat CRS (DTRS) one year after surgery (6).

The involvement of multiple immune cells and inflammatory mediators in the pathogenesis makes CRS a highly heterogeneous disease. Depending on the local infiltration of inflammatory cells (eosinophils or neutrophils) and the level of T cell-associated cytokines, CRS can be classified into 5 endotypes (7). More than 80% of CRSwNP patients exhibit a predominantly eosinophilic infiltrate, defined as T2 endotype (8), with the highest recurrence rate at 3 years after surgery (9). The accuracy of the diagnosis, the surgeon's technical skills, and the rationality and standardization of the treatment plan also affect the treatment outcome.

FESS is a classic procedure for the treatment of CRS. Based on the theory of the ostiomeatal complex (OMC), FESS is a minimally invasive surgical treatment for CRS that improves the ventilation and drainage of the nasal cavity and/or sinuses while preserving normal structure and function as much as possible, and has been widely accepted by rhinologists. However, researchers have found that the efficacy of FESS is poor in some patients, especially those with T2 CRS, even when combined with standardized drug therapy (10). How to assess and improve treatment outcomes of T2 CRS is currently a debatable and difficult issue. Among other considerations, a validated surgical prognostic indicator is important for rhinologists when developing treatment strategies.

In the current study, transcriptomic data from the Gene Expression Omnibus (GEO) database of CRS patients were screened using various bioinformatic algorithms. Hub eotaxins (CCL13 and CCL26) associated with CRS were selected for this clinical retrospective study. A total of 115 CRS patients who underwent FESS in our department over the past 2 years were enrolled and followed-up. Preoperative serum CCL26 levels demonstrated excellent diagnostic accuracy for CRSwNP. Notably, higher preoperative CCL26 concentrations were significantly associated with poor postoperative symptom improvement. This study lays the clinical foundation for using CCL26 as a prognostic biomarker after CRS surgery and provides rhinologists with a theoretical basis for developing comprehensive treatment strategies for DTRS.

## Material and Methods

### Study design

**Figure 1:**
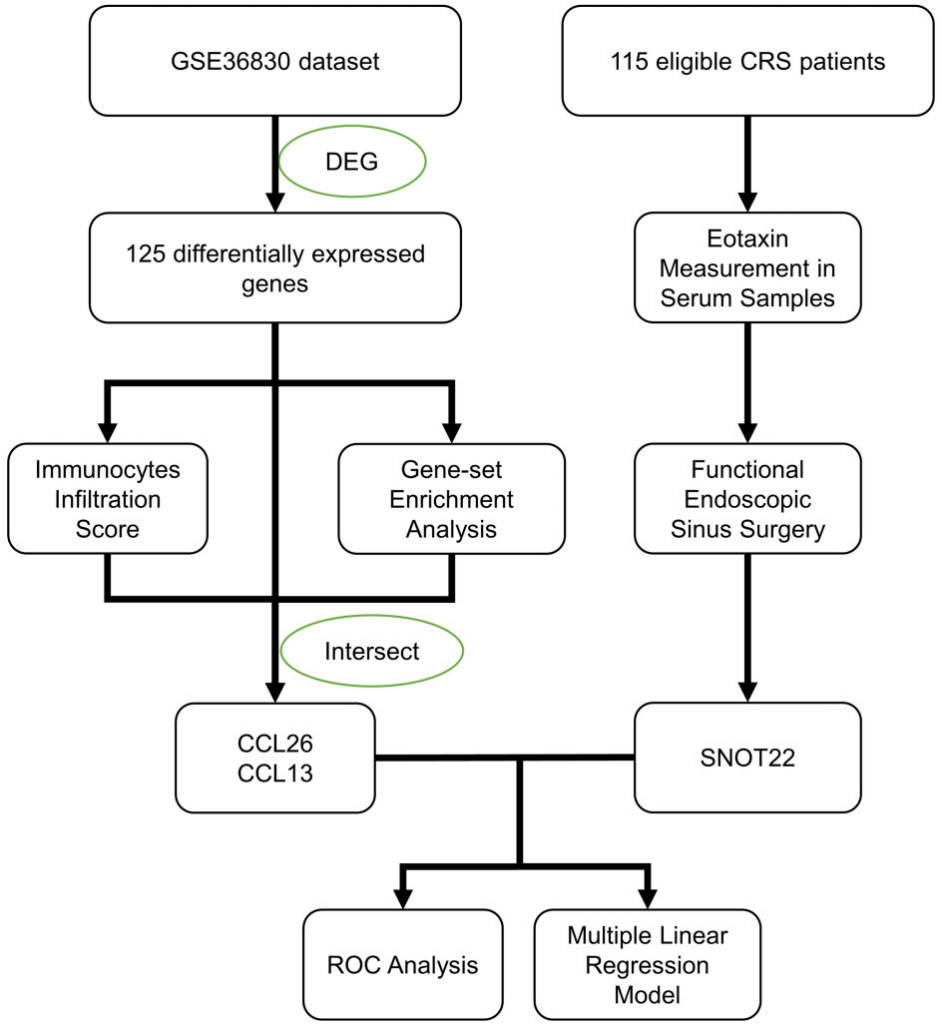
Flowchart of this study. CRS: chronic rhinosinusitis; DEG: differentially expressed genes. ROC: receiver operating characteristics.

This study employed an integrated approach combining transcriptomic bioinformatics with clinical biomarker validation. Using the GSE36830 dataset, we identified differentially expressed genes (DEGs) and selected CCL26 and CCL13 as candidate biomarkers. Serum levels of these eotaxins were then quantified preoperatively in CRS patients (n=115), with concurrent evaluation of CRS-related symptoms using the SNOT-22 questionnaire. Diagnostic performance was assessed via receiver operator characteristic (ROC) curve analysis, while prognostic utility was evaluated through multiple linear regression modeling of ΔSNOT-22 (Figure 1).

### Source of sample data

Transcriptome data from the GSE36830 project were downloaded from the GEO database website (https://www.ncbi.nlm.nih.gov/geo/). Samples of the uncinate tissues were selected for gene sequencing, including six CRSsNP (GSM902686 - GSM902691) samples, six CRSwNP (GSM902692 - GSM902697) samples, and six control (GSM902680 - GSM902685) samples. The sequencing platform used was the GPL570.

### Clinical follow-up of patients

Clinical information was collected from CRS patients who underwent FESS in our department from January 1, 2019, to December 31, 2020 (Supplementary Table S1). The inclusion criterion was a postoperative pathological diagnosis of chronic rhinosinusitis, which could avoid preoperative misdiagnosis. The exclusion criteria were: a) co-morbid psychiatric disorders. The self-assessments of these patients were inaccurate, which may affect the reliability of the SNOT22 scale results, and mental illnesses may exacerbate the subjective perception of nasal symptoms (11); b) previous history of malignancy. Our study focused on inflammatory factors closely related to malignant tumors, specifically eotaxins (12); c) previous history of immunodeficiency (13); d) intravenous use of glucocorticoids from 1 month before to 3 months after surgery (14); e) pregnancy status (15), and f) concomitant acute infectious disease (16). All of these conditions may influence the expression level of eotaxins. The surgery was performed strictly in accordance with CPOS-2018. The SNOT22 scale questionnaire was administered 1 week before surgery (preoperative SNOT22) and 3 months after surgery (postoperative SNOT22), and included five major categories: nasal symptoms, extra-nasal symptoms, ear/facial symptoms, psychological dysfunction, and sleep disturbance. Each category contained several specific symptom descriptions, each scaled from 0 to 5 points. Symptom improvement was quantified using ΔSNOT-22, calculated as preoperative minus postoperative scores, where positive values indicated better outcomes.

### DEG analysis

To analyze genes related to nasal polyps (NP) in patients with CRS, Package ‘Limma’ (3.52.1) in R software was used to analyze DEGs between CRSsNP and CRSwNP samples. Briefly, a design matrix was constructed to model experimental conditions (CRSwNP and CRSsNP). Linear models were fitted to the expression data using ‘lmFit', followed by empirical Bayes moderation with ‘eBayes’ to stabilize gene-wise variance estimates. Specific contrasts between groups were defined using ‘makeContrasts'. Adjusted P-value was analyzed using the Benjamini-Hochberg (BH) method. DEGs were identified by applying the adjusted P-value and the log2 fold change (FC) by ‘topTable'. The DEG filtering conditions were adjusted P-value <0.05 and |Log_2_FC| ≥1. The results were plotted as a heat map and volcano map using the packages of ‘pheatmap’ and ‘ggplot2', respectively.

### Immunocyte infiltration score

Package ‘CIBERSORT’ (0.1.0) in R software was used to estimate the infiltration score of 22 immune cells in every sample. The ‘pheatmap', ‘ggplot', and ‘ggpubr’ packages were used for graphical visualization. The immunocyte scores were compared between the two groups using P<0.05 as the threshold by Kruskal-Wallis rank test.

### Gene-set enrichment analysis

The ‘GSEA’ (4.2.3) software was used for Gene Ontology (GO) biological process enrichment analysis in MSigDB (7.5.1) database, and the entry threshold was set at the adjusted P-value <0.05 and enriched genes >5. ‘GOBP_EOSINOPHIL_CHEMOTAXIS’ and ‘GOBP_EOSINOPHIL_MIGRATION’ enrichment results were plotted as GSEA line graphs. The hub genes enriched in eosinophil chemotaxis and migration were intersected with differentially expressed genes. The ‘VennDiagram’ (1.7.3) package in R software was used to identify the intersecting genes and profile a Venn diagram.

### Eotaxin measurement in serum samples

Fasting venous blood was collected from the enrolled patients before surgery, and centrifuged at 1,000 *g* at 4°C for 15 min. The supernatant was stored in a refrigerator at -80°C. The expression levels of CCL26 and CCL13 in peripheral serum were measured by ELISA in strict accordance with kit instructions (Ref #EK1150S and #EK167S), purchased from Multi Sciences (LIANKE) Biotech Co., Ltd. (China).

### Ethical statement

The study was performed in accordance with the Declaration of Helsinki’s Good Clinical Practice guidelines and was approved and registered by the Ethics Committee of our hospital (approval number LJTSYXZX-LL-20180312). All questionnaires and sample collections were conducted on the premise of patients’ full disclosure of their interests in the study.

### Statistical analysis

In this study, all data are reported as means±SD. The ΔSNOT-22 was assessed using Shapiro-Wilk and Kolmogorov-Smirnov tests. In the comparison of the two groups, the *t*-test was used for quantitative data and the chi-squared test was used for qualitative data, with F and χ^2^ representing statistical data, respectively. Variables related to NP were analyzed using univariate and multivariate logistic regressions. The diagnostic value was evaluated using the ROC curve, and the area under the curve (AUC) value was calculated. The pairwise comparison of AUCs was performed using the ‘pROC’ R package with the ‘Venkatraman’ method. Multiple linear regression models were constructed to explore the relationship between SNOT22 and preoperative dependent variables. P<0.05 was considered statistically significant.

## Results

### CCL26 and CCL13 were up-regulated in the CRSwNP group

**Figure 2:**
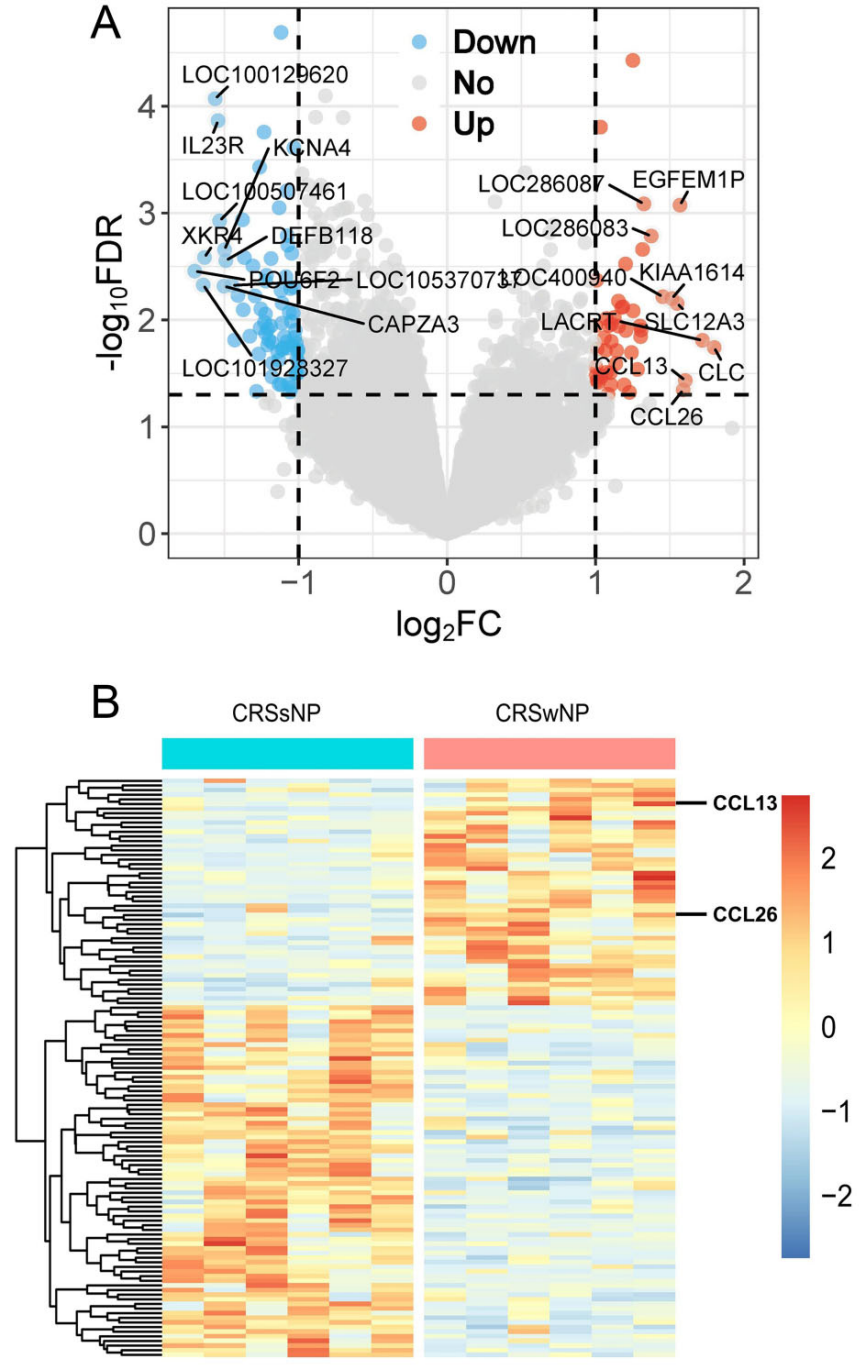
Volcano plot (**A**) and heat map (**B**) of differentially expressed genes of GSE36830. CRSsNP: chronic rhinosinusitis without nasal polyps; CRSwNP: chronic rhinosinusitis with nasal polyps.

Transcriptomic analysis identified 125 DEGs between CRSsNP and CRSwNP groups (49 upregulated, 76 downregulated). The volcano plot (Figure 2A) revealed a clear molecular distinction between subtypes, with coherent overexpression of both chemokines in CRSwNP. Similarly, row-normalized heatmap analysis (Figure 2B) confirmed significant upregulation of CCL26 and CCL13 in CRSwNP compared to CRSsNP. However, CCL26 expression had no significant difference between the control group and CRSsNP group (P=0.3528) or CRSwNP group (P=0.1495) (Supplementary Figure S1).

### Resting dendritic cells and eosinophils were significantly higher in the CRSwNP group

**Figure 3:**
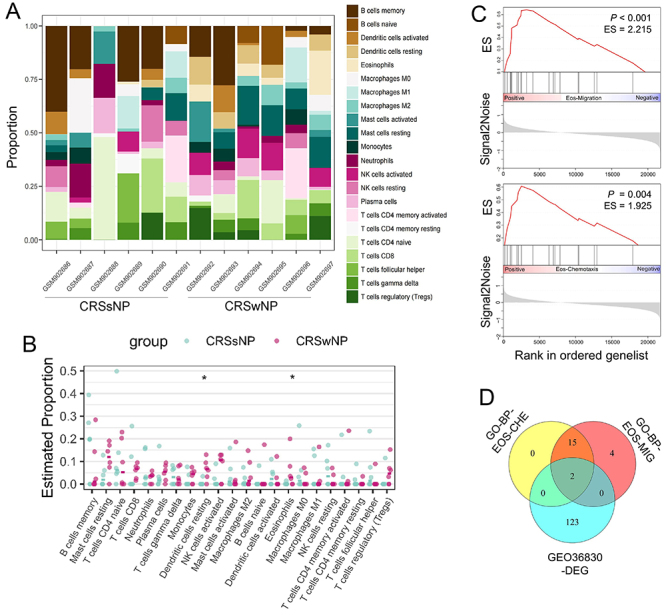
Screening core genes of CRSwNP based on the proportion of immune infiltrating cells, GSEA enrichment analysis, and DEGs. **A**, Infiltration landscape of 22 immune cell types across samples. **B**, Quantitative analysis. **C**, Functional enrichment analysis. **D**, Intersection analysis of deferentially expressed genes and enriched biological processes identified CCL26 and CCL13 as hub eotaxins. CRSsNP: chronic rhinosinusitis without nasal polyps; CRSwNP: chronic rhinosinusitis with nasal polyps.

The infiltration landscape of 22 immune cell types across samples is depicted as a stacked bar chart (Figure 3A). Quantitative analysis (Figure 3B) revealed significantly elevated proportions of resting dendritic cells and eosinophils in CRSwNP compared to CRSsNP (dendritic cells: P=0.019; eosinophils: P=0.035). Given the established pathogenic role of eosinophils in CRS, we further investigated the mechanisms underlying their enhanced infiltration.

### Biological process enrichment and core gene screening

Functional enrichment analysis (GSEA) revealed significant upregulation of eosinophil migration and chemotaxis pathways in CRSwNP (nominal P<0.001 and P=0.004, Figure 3C). These pathways contained eosinophil-recruitment genes (10 migration-related, 7 chemotaxis-related) enriched at the leading edge of the GSEA curve. Intersection analysis of DEGs and enriched biological processes identified CCL26 and CCL13 as hub eotaxins (Figure 3D), validating their mechanistic role in eosinophil trafficking.

### Serum CCL26 was an ideal biomarker of CRSwNP

**Figure 4:**
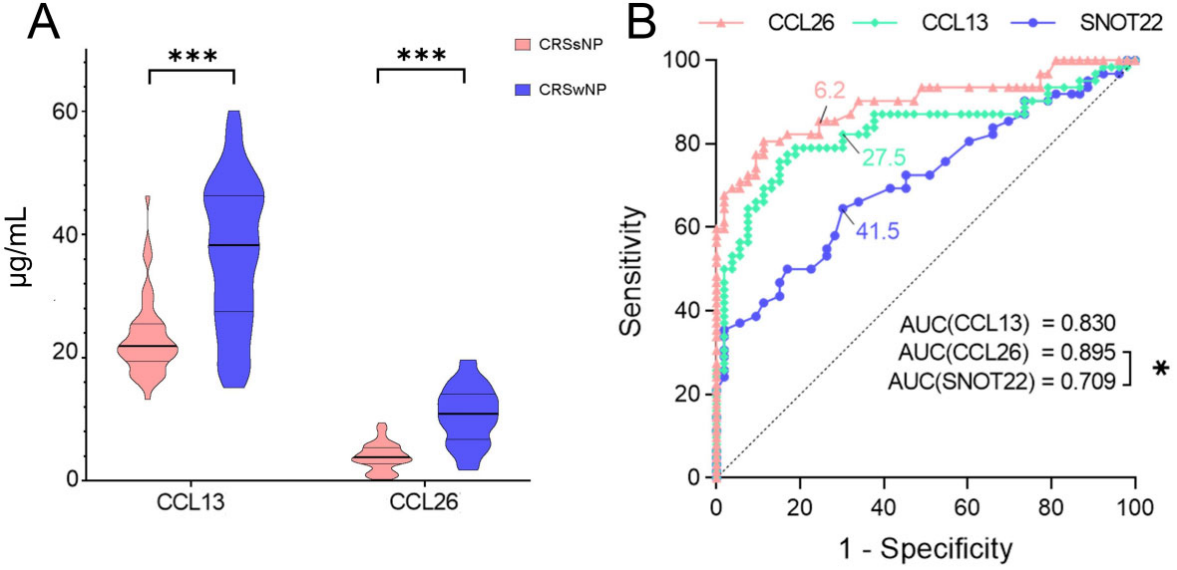
The concentrations and diagnostic value of CCL26 and CCL13 in 115 cases of follow-up of patients with chronic rhinosinusitis with nasal polyps (CRSwNP). Data are reported as means and SD. ***P<0.001; Student’s *t*-test. CRSsNP: chronic rhinosinusitis without nasal polyps.

The cohort of 115 CRS patients was stratified into CRSsNP and CRSwNP groups based on postoperative histopathological confirmation of NP. Apart from demographic variables (age, sex, smoking status), the preoperative serum CCL13 and CCL26 levels and preoperative SNOT-22 scores were analyzed as independent predictors of NP (Table 1). Compared with CRSsNP patients, the CRSwNP group exhibited significantly elevated preoperative serum concentrations of both CCL13 and CCL26 (P<0.001, Figure 4A). Both biomarkers demonstrated high diagnostic accuracy for NP, with CCL26 showing superior discriminatory capacity (AUC=0.895 [95%CI: 0.837-0.954]) compared to CCL13 (AUC=0.830 [95%CI: 0.751-0.908]) (Figure 4B).

**Table 1:** Univariate and multivariate logistic regression of nasal polyps in 115 followed-up chronic rhinosinusitis patients.

Clinical characteristics	Univariate Logistic			Multivariate Logistic		
	B	OR	P	B	OR	P
Age	0.013	1.013	0.415	0.014	1.014	0.612
Sex (male)	0.113	1.120	0.765	0.049	1.050	0.935
Smoke (Yes)	0.272	1.312	0.504	0.126	1.135	0.838
CCL13	0.148	1.159	<0.001	0.139	1.149	<0.001
CCL26	0.335	1.398	<0.001	0.211	1.235	0.004
Preoperative SNOT22	-0.048	0.953	0.002	-0.096	0.909	<0.001

CCL: C-C Motif Chemokine Ligand, SNOT: sinonasal outcome test.

### CCL26 as a reliable prognostic biomarker of ΔSNOT22 score from surgery

Patient-reported quality of life, measured by ΔSNOT-22 scores, served as the primary subjective outcome for CRS surgical success. Multiple linear regression confirmed a significant relationship between ΔSNOT-22 and preoperative serum CCL26 levels. Preoperative SNOT-22 score and NP status, with data meeting assumptions of independence and homoscedasticity, are shown in Table 2 (P<0.001). This established serum CCL26 as an independent prognostic biomarker for postoperative symptom improvement.

**Table 2:** Multiple linear regression of ΔSNOT-22 (sinonasal outcome test).

Variables	Unstandardized B (95%CI)	Standardized β	t	P
(Constant)	27.895 (24.227, 31.563)		15.070	<0.001
Pre_SNOT22	0.173 (0.098, 0.247)	0.224	4.578	<0.001
CCL26	-1.212 (-1.456, -0.968)	-0.564	-9.854	<0.001
Nasal polyps^a^	-7.144 (-9.674, -4.614)	-0.335	-5.596	<0.001

^a^0: chronic rhinosinusitis without nasal polyps (CRSsNP); 1: chronic rhinosinusitis with nasal polyps (CRSwNP). CCL: C-C Motif Chemokine Ligand.

## Discussion

The pathogenesis of NP is complex. Microscopy shows mucosal thickening, glandular hyperplasia or squamous epithelial hyperplasia, often covered with pseudostratified epithelium and goblet cells, and a large number of inflammatory cells, predominantly eosinophils (17). Therefore, CRS is further categorized as eosinophilic CRS (ECRS) and non-eosinophilic CRS (NECRS). According to a multicenter large-scale epidemiological survey, ECRS is definitively defined when the eosinophil count in nasal mucosa is greater than or equal to 70 eosinophils/HP (magnification, ×400) (18). The eosinophilic role in the development of NP is gradually being discovered in eosinophil chemotaxis and migration (19). Interleukin (IL)-5 is a critical chemokine in the promotion of the expression of intercellular adhesion molecule (ICAM) in eosinophils, which enhances the adhesion of eosinophils to the vascular endothelium and tends to increase the selective migration of eosinophils from the peripheral blood into mucosal tissue (20). In addition, TGF-α is highly expressed in the nasal tissue of ECRS patients and is deeply involved in mucus hypersecretion (17). Furthermore, macrophage inflammatory protein-1 (MIP-1), tumor necrosis factor-α (TNF-α), and CCL13 are reported in ECRS (21-23).

Although this study did not identify all previously reported CRS-associated eotaxins, extracting the CCL26 and CCL13 genes helped compensate for the screening and validation of eotaxin at the transcriptome level. CCL26, a classic chemokine also known as eotaxin-3/ C-C motif ligand 26, has been the subject of a few recent studies in this field. Some researchers have analyzed gene microarray data of the nasal cavity and ethmoid sinus lavage fluid. Compared to normal patients, CCL26 is more than 3-fold and significantly elevated in CRSsNP with the type 3 (T3) endotype (24). In a case-control study of CRSsNP, it has been found that the plasma CCL26 levels are significantly higher in the high-eosinophil mucosal infiltration group (25). Studies have found that serum leptin levels were correlated with CCL26 mRNA expression in NP tissue of CRSsNP (26), which can be inhibited by proton pump inhibitors (PPIs) directly in IL-16-dependent nasal epithelial cells (27). In addition, experts have discovered that CCL26 promotes eosinophilic NP through negative regulation of the STING pathway. Nevertheless, the exact molecular biological mechanism and its relationship with prognosis and treatment remain unclear.

Clinical treatment options for CRSwNP include drug therapy, surgical treatment alone, and a combination of surgery and medication. Despite adequate, standardized pharmacological and surgical treatments, a considerable number of patients still do not achieve satisfactory results. Due to serious methodological limitations, reporting bias, indirectness, and imprecision, the evidence does not show that one treatment is superior to another (28). Personalized biologic therapy targeting the patient's immune profile, combined with appropriate surgery based on their nasal sinus structure, may be a practical strategy to achieve better outcomes.

The new insight of biologics targeting T2 has paved the way for treating refractory CRS, especially ECRS. Clinical studies have shown that in refractory CRSwNP, mepolizumab, a humanized anti-IL-5 monoclonal antibody, significantly improves polyp scores and reduces the need for surgery (29-31). However, tissue eosinophils are reduced, and the local inflammatory microenvironment may undergo feedback regulation of T2 inflammatory loops with prolonged use. Therefore, the therapeutic effect of mepolizumab is not permanent (32).

In addition, it is worth mentioning that the immune infiltration analysis in our study also showed significantly different levels of dendritic cell infiltration between CRSwNP and CRSsNP. The role of dendritic cells as antigen-presenting cells in the pathogenesis of CRSwNP has been less concerning than that of eosinophils. A recent study found that both direct smoking and second-hand smoke increased dendritic cells of monocyte origin in patients with CRSwNP, and *in vitro* experiments confirmed that these cells secrete large amounts of immunosuppressive agents such as IL-10 and INF-γ (33). The role of dendritic cells in the pathogenesis of CRSwNP needs to be further researched.

This study had several limitations that warrant acknowledgment. First, the transcriptomic analysis relied on the GSE36830 dataset (n=24), whose limited sample size reduces the power to detect subtle gene expression changes and reflects broader challenges in accessing CRS datasets with paired clinical-transcriptomic data. Second, our clinical validation cohort (n=115) limits statistical power for subgroup analyses and generalizability, increasing the risk of type II error for nuanced biological associations. To address these issues, future work should: a) integrate multi-cohort transcriptomics (e.g., GEO/EGA repositories) to increase discovery power; b) establish prospective multi-center cohorts with standardized phenotyping; c) employ longitudinal multi-omics designs (proteomics/metabolomics) to validate mechanisms; and d) apply advanced computational methods (e.g., covariate-adjusted batch correction) to mitigate confounders. These strategies will enhance reproducibility, clarify CCL26's role in CRSwNP heterogeneity, and accelerate clinical translation.

## Conclusions

Given its dual diagnostic-prognostic value (AUC=0.895 for CRSwNP; β=-0.564 for ΔSNOT-22), CCL26 measurement could optimize patient selection for FESS and guide postoperative management.

## Data Availability

The datasets used and/or analyzed during the current study are available from the corresponding author on reasonable request.

## Supplementary Materials

Click to view [pdf].
