# Supplementary material for: Prognostic value of preoperative CCL26 serum levels in the surgical treatment of chronic rhinosinusitis
Source: Braz J Med Biol Res. 2026 Aug 7;59:e14736. doi: 10.1590/1414-431X2026e14736 (PMC13450811; doi:10.1590/1414-431X2026e14736)

Table S1. Follow-up data for 115 chronic rhinosinusitis patients who underwent functional endoscopic sinus surgery.

ID	Age (y)	Sex	Smoke	CCL13 (μg/mL)	CCL26 (μg/mL)	NP	Preoperative SNOT22	Postoperative SNOT22	ΔSNOT-22
1	52	1	0	17.2	5.7	0	40	7	33
2	46	1	0	20.6	6	0	31	5	26
3	58	0	1	24.3	5.1	0	67	40	27
4	34	0	0	39.1	19.6	1	52	60	-8
5	33	0	1	17.3	6	1	24	7	17
6	63	0	1	32.3	14.8	1	55	44	11
7	61	0	1	20	3.1	0	35	3	32
8	38	0	1	13.2	3.2	0	48	8	40
9	52	0	1	22.3	3.2	0	31	6	25
10	66	1	1	21.5	3.5	0	43	13	30
11	46	0	1	57.3	16.8	1	67	65	2
12	57	1	1	20.5	3.5	0	46	14	32
13	45	0	1	19.3	4.3	0	36	6	30
14	31	0	1	15.6	1.1	0	36	4	32
15	56	1	1	30.4	13.6	1	35	20	15
16	50	0	1	60.1	3.9	1	47	24	23
17	48	0	1	38.6	15.8	1	20	14	6
18	38	0	0	36.2	7.1	0	49	20	29
19	52	0	1	22.2	5.7	0	37	4	33
20	61	0	1	29.2	4.8	0	43	12	31
21	42	0	1	37.1	16.5	1	28	16	12
22	80	0	1	19.7	2.4	1	18	5	13
23	61	1	1	19.6	3.9	1	19	9	10
24	58	0	1	25.2	4.1	0	70	42	28
25	42	0	1	50.5	4.8	1	45	17	28
26	51	0	1	29.1	13.6	1	40	28	12
27	46	1	1	21.9	1.7	0	41	7	34
28	48	1	1	27.4	3.8	0	56	29	27
29	67	0	0	45.8	9	1	39	21	18
30	67	0	1	46.4	6.3	1	40	15	25
31	48	1	1	17.2	6.1	0	48	15	33
32	43	1	1	26	1.7	1	22	10	12
33	36	0	1	26.9	10.9	1	32	12	20
34	21	0	1	20.1	1.3	0	51	12	39
35	43	0	1	41.4	12.4	1	26	7	19
36	55	1	1	45.2	12.9	1	20	6	14
37	68	0	0	55.1	6.7	1	18	9	9
38	63	0	0	20	0.8	0	59	20	39
39	54	0	1	22.7	4.1	0	54	26	28
40	50	0	0	16.8	5.3	1	22	7	15
41	47	0	1	30.8	3.1	0	63	37	26
42	22	1	1	49.7	8.9	1	28	11	17
43	29	0	1	27.7	11.2	1	26	7	19
44	41	0	0	41.3	8.7	1	30	13	17
45	42	1	1	46.3	17.7	1	49	50	-1
46	50	0	1	57.7	10.4	1	42	23	19
47	31	0	0	17.1	2.4	1	7	5	2
48	44	0	1	48.3	1.7	1	32	7	25
49	48	1	1	21.9	1.2	0	56	21	35
50	54	1	0	21.2	4.4	0	49	22	27
51	47	0	1	38.9	9.2	1	48	27	21
52	55	1	1	17.6	5.2	0	42	5	37
53	56	0	0	29.4	8.7	0	22	8	14
54	48	1	1	31	16.2	1	33	27	6
55	60	1	0	49.6	14.2	1	60	47	13
56	43	0	0	22.5	5	0	55	22	33
57	61	0	0	24.5	12.5	1	41	18	23
58	45	0	1	21.7	0.2	0	37	0	37
59	50	0	0	16.3	0.6	0	65	28	37
60	30	1	1	36.4	15.3	1	45	32	13
61	44	1	0	17.6	5.7	0	52	17	35
62	42	1	1	36.6	9.4	0	30	5	25
63	54	0	1	44	9.7	1	43	25	18
64	61	1	1	51	15.1	1	63	57	6
65	41	1	1	48.6	4.8	1	37	8	29
66	54	1	0	37.9	11.5	1	51	35	16
67	44	1	1	23.1	8.3	1	27	5	22
68	62	1	0	47.7	14.5	1	48	38	10
69	61	1	1	23.9	2.1	0	46	18	28
70	55	1	1	44.7	6.9	1	37	16	21
71	43	0	0	31.5	5.5	0	36	5	31
72	31	1	1	16	2.8	0	43	7	36

73	60	1	1	44	9.5	1	20	5	15
74	50	0	1	25.4	4.3	0	49	17	32
75	43	0	0	18.1	5	1	17	3	14
76	22	1	0	17.7	6.1	0	33	1	32
77	5	0	1	21.1	0.4	0	44	6	38
78	36	0	1	38.5	7.7	0	57	29	28
79	41	1	0	34.2	10.9	1	43	28	15
80	67	0	1	36.5	15.5	1	34	29	5
81	61	1	1	30.6	19.3	1	20	24	-4
82	38	1	1	29.3	12.9	1	31	10	21
83	42	1	1	24.3	3.9	0	30	2	28
84	49	0	0	25.6	4.4	0	44	13	31
85	53	0	0	22.9	7.3	1	4	1	3
86	33	0	1	35.7	9	1	67	47	20
87	42	1	1	47.9	12.7	1	41	29	12
88	49	1	1	19.3	3.5	0	48	11	37
89	56	1	0	39	10.5	1	41	26	15
90	38	1	0	45	16.7	1	59	54	5
91	42	0	1	18.5	19.4	1	29	22	7
92	39	0	1	46.4	6.7	1	48	21	27
93	32	0	0	38.6	6.4	1	34	7	27
94	48	0	1	17.7	3.2	0	42	11	31
95	52	0	1	25.5	1.6	0	54	27	27
96	53	0	1	27.6	14	1	47	27	20
97	31	1	1	20.9	6.5	0	65	35	30
98	54	1	1	22.6	3.6	0	33	6	27
99	52	1	1	24.4	5.3	1	19	6	13
100	41	1	1	21.2	3	0	63	30	33
101	50	0	1	24.7	0.9	0	43	6	37
102	40	0	1	44.7	13.7	1	41	27	14
103	44	0	0	46.2	8.5	0	52	29	23
104	61	0	0	52.5	12.2	1	53	35	18
105	41	1	1	15.1	8.8	1	50	21	29
106	31	0	0	19.8	0.6	0	54	16	38
107	45	1	0	42.4	11	1	53	39	14
108	43	0	1	35.1	12.8	1	35	20	15
109	50	1	1	26.8	4.3	0	46	14	32
110	68	1	0	25.7	2.9	0	51	24	27
111	52	0	1	16.8	3.5	0	47	8	39
112	53	0	1	23.2	5	0	47	17	30
113	44	0	1	23.6	11.4	1	16	4	12
114	50	0	0	33.7	10.4	1	18	3	15
115	41	1	0	19.5	2.7	0	32	2	30

In "sex" column 1: Male, 0: Female. In "smoke" column 1: History of smoking, 0: No smoking history. In "CCL13" and "CCL26" column, the number represents eotaxin level in serum of cases collected before surgery. In "NP" (nasal polyps) column, 1: chronic rhinosinusitis with nasal polyps (CRSwNP), 0: chronic rhinosinusitis without nasal polyps (CRSsNP). Preoperative SNOT22: SNOT-22 score before surgery after admission. Postoperative SNOT22: SNOT-22 score tested 3 months after operation. ΔSNOT-22: Preoperative SNOT22 – Postoperative SNOT22.

Figure S1. CCL26 expression in the CRSsNP and CRSwNP groups compared with the control group. CRSsNP: chronic rhinosinusitis without nasal polyps; CRSwNP: chronic rhinosinusitis with nasal polyps.

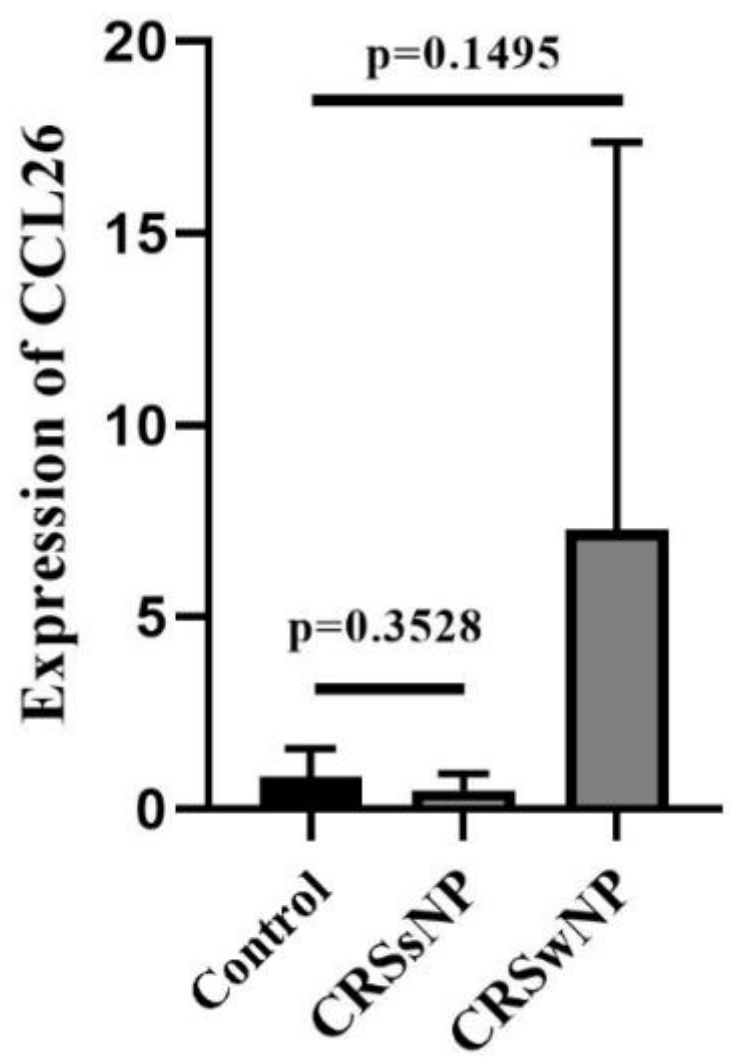

Supplement: Supplementary file 1 [file 1414-431X-bjmbr-59-e14736-suppl.pdf]
